# Welfare Workers in Industry

**Published:** 1920-12-11

**Authors:** 


THE PUBLIC WELL-BEING—(*continued*).

### Welfare Workers in Industry.

HUMANISING METHODS.

We have received a full and interesting account of the discussions at the recent Conference of Welfare Supervisors at Balliol College, Oxford. Some very able lectures were delivered, and the event served to show not only the important part which these supervisors are already playing in improving the conditions of industry—humanising work, reducing fatigue, changing the relations between masters and men—but the still more valuable part which welfare work seems to be destined to play in the future. At present many of the supervisors are, no doubt, working in discouraging circumstances. Welfare work, as Mr. Arthur Henderson, one of the great Labour leaders, frankly acknowledges, is still regarded with some suspicion by the organised workers, " but the hostility arises from a misunderstanding of the purpose and value of the work which welfare workers are trying to do in face of many difficulties. . . . Their work becomes increasingly necessary as the reorganisation of industry is carried further, and working-class antagonism will disappear when we have surmounted, as I am confident we shall surmount, the problems that engage the anxious attention of employers and workpeople at the present time." Mr. J. R. Clynes, another influential trade union leader, has faith in the success of this form of service, which, he says, is absolutely necessary " for the betterment of large masses of employees who in previous years were left entirely without the human assistance and advice which the Welfare Society can now provide." Legislation affecting the work of these welfare experts will undoubtedly have to be passed as time goes on, and the welfare supervisor will certainly fill a very big place in industry. He does not, so to speak, belong to the management any more than to the workpeople. He seems " to belong to a cause," though to-day he is paid by the employer.

### The Human Personality.

The best statement of the essential considerations in the conduct of industry, from the human point of view, was put forward in a first-rate paper by Mr. Henry Clay. Scientific management, he pointed out, is simply a further systematisation, and, by itself, dehumanisation (if there is such a word) of industry. It is just because industry becomes more and more standardised, the scale of production larger and larger, the scope and range of collective agreements and statutory regulations wider and wider, that the need arises for a special effort to interpose the activities and influence of human personality. Industry tends to become impersonal; yet the parts of it remain persons, and their relations will not be satisfactory unless their personality is considered. The welfare supervisor has the opportunity of restoring to industry some of the personal consideration that was forthcoming, without special thought or provision, when the scale of industry was smaller, and the relations of employers and their workpeople more intimate.

No amount of regulation from outside, it may be pointed out in connection with these comments, will do away with the need for personal explanations. The most carefully elaborated wages agreement or order under a statute will leave some loopholes for misunderstanding, which will lead to unnecessary suspicion and ill-feeling. The points of view of management and operative are so different, and the work of direction and planning tends to become so far removed from the actual execution of work in the shops, that the most necessary and innocent changes may be misunderstood. And, conversely, the management may quite miss the true reason for some unrest among the men. There is room for someone, in touch with both, who can offer explanations where necessary and remove misunderstanding. Little grievances, that might have been removed without cost to anyone except a little trouble, have a habit of rankling and accumulating until there is an explosion.

On the health side there are innumerable ways in which the supervisors can render vital service, especially at a time when the problem of reducing fatigue in industry is becoming one of general application.

### Reducing Fatigue.

Appropriate to this subject is an article appearing in the *Journal of Industrial Welfare*, which discusses practical methods of reducing fatigue. Investigations in the science of fatigue are going on everywhere. The Society of Industrial Engineers has established an international committee for the investigation of problems of eliminating unnecessary fatigue in the industries. This committee is collecting data on fatigue elimination in all countries, and is glad to co-operate with all interested in the subject, to receive into its membership a representative of any body interested in the subject, and to place its findings at the disposal of everyone. Data are being collected in the fields of psychology, physiology, psychiatry, and the allied sciences.

If every member of the community, the authors of the article declare, would study his own activities for one day, and try to carry out the improvements that are bound to suggest themselves, the solution of the problem of eliminating unnecessary fatigue would be assured.

